# Prevalence and Predictors of Environmental Tobacco Smoke Exposure among Adolescents in Cambodia

**DOI:** 10.4103/0970-0218.62556

**Published:** 2010-01

**Authors:** Emmanuel Rudatsikira, Seter Siziya, Adamson S Muula

**Affiliations:** Departments of Biostatistics and Epidemiology and Global Health, School of Public Health, Loma, Linda University, California, US; 1 Community Medicine, School of Medicine, University of Zambia, Lusaka, Zambia; 2 Community Health, College of Medicine, University of Malawi, Blantyre, Malawi

**Keywords:** Adolescent health, Cambodia, environmental tobacco exposure, smoking, tobacco

## Abstract

**Objective:**

To estimate the prevalence and predictors of environmental tobacco smoke (ETS) exposure among nonsmoking adolescents in Cambodia.

**Materials and Methods:**

Analysis of data from the Global Youth Tobacco Survey (GYTS) conducted in 2003 in Cambodia. Data were analysed to obtain the prevalence of ETS exposure at home and elsewhere by age and gender. Logistic regression analysis was conducted to assess the association between ETS and gender, age, smoking status of parents and friends.

**Results:**

67.1% (64.0, 70.0) males and 67.4% (64.2, 70.5) females reported being exposed to ETS either at home or elsewhere. Adolescents who had one or both smoking parents had a more than three times the odds of ETS exposure at home (OR = 3.71; 95% CI (3.02, 4.57)). Those who had smoking friends were more likely to be exposed to ETS both at home and outside home (OR = 1.74; 95% CI (1.36, 2.24)). The overall proportion of adolescents exposed to ETS outside home was higher than those exposed at home (*P* < 0.001), suggesting that exposure in public areas was the main form of ETS among adolescents in Cambodia.

**Conclusions:**

Exposure to ETS is high among adolescent in Cambodia, which indicates an urgent need for specific measures, policies and regulations to protect nonsmoker Cambodian adolescents both within and outside home.

## Introduction

Exposure to ETS is the third leading preventable cause of death worldwide.(1) In children, ETS is associated with asthma,(2) bronchitis and pneumonia.(3) Results from a study conducted in Sweden indicate that ETS in childhood is positively associated with increased prevalence of asthma in adults.(4) A study conducted in Thailand found that ETS exposure increases nine times the risk of tuberculosis among children, who have received the Bacillus Calmette-Guerin (BCG) vaccine against tuberculosis.(5) In the United States, ETS exposure was found to be associated with the metabolic syndrome among teenagers.(6) Abilities such as reading and visual spatial reasoning skills are negatively impacted by exposure to ETS.(7)

Lifetime exposure to ETS is reported to be associated with chronic obstructive pulmonary disease in adults.(2) It is of interest to estimate the prevalence and predictors of ETS exposure among adolescents. The Global Youth Tobacco Collaborative project has conducted most of the studies aimed to assess the prevalence of tobacco use among school-going adolescents globally.(8) Prevalence estimates of ETS in several countries among the GYTS study participants have been reported before by the Global Tobacco Surveillance Collaborative Group (GTSS).(9) However, we are unaware of any studies that have studied the predictors of ETS among adolescents in Cambodia. In this study, we report the prevalence of ETS exposure and predictors of exposure among school-going adolescents in Cambodia.

## Materials and Methods

### Study design

This was a cross-sectional study conducted among 13-15-year old school-going adolescents in Cambodia in 2003. A two-stage cluster sampling approach was used in which the primary sampling units were schools. In the second stage, eligible classes within the school were randomly selected. All students within the selected schools were eligible to participate in the study.

### Data collection

Study participants completed a modified GYTS questionnaire according to the standard procedures of the GYTS.(10) The GYTS questionnaire provides a set of standardized core questions. Country teams may also include a limited number of questions to collect information that may be specific to their area. Completion of the questionnaire is estimated to take about 40 min.

Permission to conduct the study was obtained from the Ministry of Education. Eligible students were informed that they were free not to participate. Questionnaires were completed anonymously.

### Statistical analysis

Responses to the following questions were used in the analysis: Do you smoke? Do your parents smoke? Do any of your closest friends smoke cigarettes? During the past 7 days, on how many days have people smoked in your home, in your presence? During the past 7 days, on how many days have people smoked in your presence, in places other than in your home? ETS exposure was defined as having had people smoke in one's presence on day or more in the last 7 days. Data were analysed using SUDAAN version 9.0 (Research Triangle Institute, Research Triangle Park, North Carolina, United States). Analyses conducted include the prevalence of ETS and association between ETS exposure and age, gender, smoking status of parents and closest friends.

## Results

### Socio-demographic characteristics of study participants

Of the 1956 adolescents who participated in the Cambodian GYTS in 2003, 94.6% were non-smokers. Table 1 presents selected demographic characteristics of the 1851 nonsmoking Cambodian adolescents. Most of the sample (median age: 15 years) was male (61.9%), 16 years old or older. (54.5%), had nonsmoking parents (51.5%) and nonsmoking friends (72.2%)

**Table 1:** Selected demographic characteristics of the Cambodian nonsmoker teenagers

	Total (%) (95% CI) (*n*)	Males (%) (95% CI) (*n*)	Females (%) (95% CI) (*n*)
All	100 (1851)	61.9 (59.7, 64.1) (997)	38.1 (35.9, 40.3) (854)
Age (years)			
≤12	10.8 (9.4, 12.3) (204)	12.2 (10.3, 14.3) (128)	8.4 (6.7, 10.5) (76)
13	7.6 (6.5, 8.9) (163)	6.0 (4.7, 7.5) (69)	10.3 (8.4, 12.5) (94)
14	9.5 (8.3, 10.8) (212)	7.4 (6.1, 9.1) (89)	12.8 (10.8, 15.1) (123)
15	17.7 (16.0, 19.5) (346)	15.6 (13.5, 18.0) (166)	21.1 (18.3, 24.1) (180)
≥16	54.5 (52.3, 56.6) (926)	58.8 (55.8, 61.8) (545)	47.5 (44.1, 50.1) (381)
One or both parents smoking	48.5 (46.2, 50.9) (1830)	50.6 (47.4, 53.8) (986)	45.1 (41.7, 48.6) (844)
Friends smoking	27.8 (25.6, 30.0) (1841)	37.3 (34.3, 40.4) (992)	12.2 (10.0, 14.8) (848)
ETS exposure	49.1 (46.7, 51.4) (1885)	60.0 (57.7, 62.3) (1872)	67.1 (64.9, 69.3)

### Prevalence and predictors of exposure to environ- mental tobacco smoke

Table 2 indicates that 67.1% of the subjects were exposed to ETS at home or elsewhere. The proportion of adolescents exposed to ETS outside home was higher than those exposed at home (60.1%; 95% CI (57.9, 62.3) and 49.1%; 95% CI (46.8, 51.4)) (*P* < 0.001). Older participants had the highest ETS exposure prevalence outside home (63.5%; 95% CI (60.2, 66.8)), while the youngest had the lowest exposure rates outside home (52.3%; 95% CI (45.5, 59.1)). No differences were found between male and female participants.

**Table 2:** Percentage of Cambodian teenagers exposed to environmental tobacco smoke at home, outside home and in both settings

	Home (%) (95% CI) (*n*)	Outside of the home (%) (95% CI) (*n*)	Both home and outside of the home (%) (95% CI) (*n*)
*P* value	*P* = 0.02	*P* = 0.009	*P* = 0.004
All	49.1 (46.8, 51.4) (1885)	60.1 (57.9, 62.3) (1872)	67.1 (64.9, 69.3) (1892)
≤12	46.1 (39.6, 52.9) (235)	52.3 (45.5, 59.1) (235)	58.6 (51.8, 65.0) (238)
13	41.8 (34.1, 49.9) (167)	55.7 (47.7, 63.4) (165)	59.6 (51.6, 67.1) (167)
14	51.2 (44.3, 58.1) (212)	58.0 (51.1, 64.7) (345)	70.8 (64.3, 76.6) (212)
15	45.2 (39.7, 50.8) (348)	57.6 (52.0, 63.0) (345)	66.1 (60.7, 71.2) (349)
≥16	51.7 (48.4, 55.0) (992)	63.5 (60.2, 66.8) (916)	69.8 (66.6, 72.8) (926)
*P* value	*P* = 0.04	*P* = 0.02	*P* < 0.01
Males	49.5 (46, 52.7) (991)	60.2 (57.1, 63.3) (985)	67.1 (64.0, 70.0) (993)
≤12	47.6 (38.5, 56.8) (127)	53.6 (44.3, 62.6) (126)	58.7 (49.4, 67.4) (127)
13	47.8 (35.8, 60.0) (69)	61.3 (48.8, 72.4) (68)	64.2 (51.8, 74.9) (69)
14	59.3 (48.5, 69.2) (89)	66.2 (55.4, 75.6) (88)	79.8 (70.1, 86.9) (89)
15	41.6 (34.0, 49.6) (166)	54.0 46.0, 61.8) (165)	62.4 (54.5, 69.7) (166)
≥16	51.0 (46.8, 55.2) (540)	62.4 (58.2, 66.6) (538)	68.7 (64.6, 72.6) (543)
*P* value	*P* = 0.02	*P* < 0.01	*P* < 0.01
Females	48.1 44.7, 51.5) (847)	60.0 (56.7, 63.3) (840)	67.4 (64.2, 70.5) (851)
≤12	42.0 (30.8, 54.0) (75)	50.0 (38.5, 61.6) (75)	58.3 (46.6, 69.2) (76)
13	35.8 (26.3, 46.7) (94)	51.6 (41.0, 62.0) (93)	54.9 (44.3, 64.1) (94)
14	43.6 (35.0, 52.6) (123)	50.4 (41.5, 59.2) (123)	62.4 (53.4, 70.6) (123)
15	48.9 (41.2, 56.7) (179)	62.2 (54.5, 69.3) (177)	70.5 (63.0, 77.0) (180)
≥16	52.7 (47.6, 57.7) (376)	65.4 (60.4, 70.1) (372)	71.8 (67.1, 76.1) (378)

Table 3 indicates that exposure to ETS among nonsmokers was strongly associated with having one or both smoking parents. For subjects whose parents were smokers, they had more than four times the odds of ETS exposure at home (OR = 4.86; 95% CI (3.55, 6.64)) and a more than two-times the odds of exposure outside of the home (OR = 2.53; 95% CI (1.87, 3.42)). Having smoking friends was positively associated with ETS exposure. Those who had smoking friends had two times the odds of ETS exposure at home (OR = 2.01; 95% CI (1.61, 2.52)) and 97% increase in the odds of ETS exposure outside home (OR = 1.97; 95% CI (1.52, 2.54)). Older age was associated with increased odds of exposure to ETS. Those aged 16 years or older had 59% increase in the odds of ETS exposure at home and 64% increase in the odds of ETS exposure outside home compared to those aged 12 years or younger (OR = 1.59; 95% CI (1.17, 2.16)) and OR= 1.64; 95% CI (1.20, 2.24)).

**Table 3:** Variables associated with exposure to environmental tobacco smoke among Cambodian teenagers - Univariate analysis

	ETS exposure at home	ETS exposure outside at home	ETS exposure both at home and outside home
			
	OR (95% CI)	OR (95% CI)	OR (95% CI)
Age (years)			
≤ 12	1.00	1.00	1.00
13	0.84 (0.55, 1.29)	1.14 (0.75, 1.75)	1.04 (0.68, 1.59)
14	1.26 (0.83, 1.81)	1.26 (0.75, 1.15)	1.72 (1.15, 2.28)
15	0.96 (0.68, 1.37)	1.24 (0.87, 1.76)	1.36 (0.96, 1.98)
≥16	1.25 (0.92, 1.70)	1.59 (1.17, 2.16)	1.64 (1.20, 2.24)
Gender			
Females	1.00	1.00	1.00
Males	1.06 (0.87, 1.28)	1.01 (0.83, 1.22)	0.98 (0.80, 1.20)
Parents smoking			
None	1.00	1.00	1.00
One or more parents smoking	4.86 (3.55, 6.64)	2.53 (1.87, 3.42)	3.08 (2.22, 4.29)
Friends smoking			
No	1.00	1.00	1.00
Yes	1.73 (1.39, 2.24)	2.01 (1.61, 2.52)	1.97 (1.52, 2.54)

Table 4 indicates that parental smoking and having smoking friends remained positively associated with ETS exposure both at home and outside home in multivariate analysis.

**Table 4:** Variables associated with exposure to environmental tobacco smoke among Cambodian teenagers - Multivariate analysis

	ETS exposure at home	ETS exposure outside at home	ETS exposure both at home and outside home
			
	OR (95% CI)	OR (95% CI)	OR (95% CI)
Age (years)			
≤ 12	1.00	1.00	1.00
13	0.94 (0.58, 1.52)	1.50 (0.95, 2.37)	1.18 (0.73, 1.91)
14	1.45 (0.94, 2.25)	1.49 (0.98, 2.27)	1.13 (0.73, 1.77)
15	0.94 (0.63, 1.39)	1.40 (0.95, 2.07)	0.90 (0.60, 1.34)
≥16	1.13 (0.79, 1.60)	1.62 (1.15, 2.27)	1.21 (0.84, 1.72)
Gender			
Females	1.00	1.00	1.00
Males	0.90 (0.73, 1.11)	0.86 (0.70, 1.06)	0.91 (0.73, 1.12)
Parents smoking			
None	1.00	1.00	1.00
One or more parents smoking	3.71 (3.02, 4.57)	2.05 (1.67, 2.52)	3.18 (2.58, 3.92)
Friends smoking			
No	1.00	1.00	1.00
Yes	1.75 (1.35, 2.27)	1.82 (1.40, 2.37)	1.74 (1.36, 2.24)

## Discussion

This study identified a strong association between parental smoking status and ETS exposure among nonsmoking adolescents both at home [OR = 4.86; 95% CI (3.55, 6.64)] and outside home (OR = 2.53; 95% CI (1.87, 3.42)). Adolescents who have smoking parents at home may have more tolerant attitudes towards ETS outside home than those without smoking parents; having smoking friends was positively associated with ETS exposure both at home and outside home. Bird *et al*. (2006) report similar findings in Mexico.(11) A previous study report suggests that adolescents who have nonsmoking friends may be better prepared to avoid ETS exposure than those with smoking friends.(12) We found that males and females had similar ETS exposure rates both at home and elsewhere (67%). These results differ from findings from Greece where males were reported to have less ETS exposure rate than females.(13) In this study, age was positively associated with ETS exposure and interestingly ETS exposure outside home was higher than at home ETS exposure. Adolescents may be exposed to ETS at school from peers smoking as well as from teachers.(14) A study report from India indicates that smoking in ‘significant others’ such as friends or parents was associated with being a smoker among the study participants.(15) This is in harmony with developmental adolescent research that suggests that as adolescents get older, socializing with other adolescents their age becomes an important formative behavior toward achieving separation and independence from their parents.

Our findings have important public health implications. The burden of disease increases considerably among nonsmokers exposed to ETS.(16) Exposure to ETS has previously been reported to be associated with higher all cause mortality and with increased mortality due cardiovascular disease,(17) cancer, especially lung cancer,(18) chronic obstructive pulmonary disease,(19) respiratory symptoms of infectious and non-infectious nature(20) and stroke.(21)

In a study conducted from Brazil and using the same survey methods as in our study, Hallal *et al*,(22) reported that factors significantly associated with cigarette smoking among adolescents were as follows: Having smoking friends and being exposed to environmental smoke outside the home. Our findings are also similar to what Mbulo had observed in the United States in 2006 that girls were significantly more likely to be exposed to secondhand smoke than in boys.(23)

Our study had several limitations. Firstly, we did not assess biomarkers of tobacco smoke exposure such as cotinine levels in study participants who reported exposure to ETS. As the study participants were asked to report past 7 day exposure, this would have been possible to detect in urine.(24) However, our study used a standardized self-report questionnaire that enables within country and cross-country comparisons of ETS exposure. Secondly, the sample was recruited from school-going adolescents and therefore is not representative of all Cambodian nonsmokers. Also data were collected from students who attended school on the day of the survey. No attempt was made to seek out those who did not attend at the day of data collection. Finally, this was a self-report questionnaire. There is therefore potential for mis-reporting by study participants.

## Conclusions

Public health interventions aimed at limiting ETS exposure among adolescents should consider both the home and the out home environment. Smoking parents should be encouraged to take precautions to not expose their children to ETS. Such precautions are reported to decrease children's ETS exposure rates by one-third and substantially improve the health of nonsmoking adolescents who live with smoking adults.(11) In accordance with the Framework Convention on Tobacco Control (FCTC) ratified by Cambodia in 2005,(25) passing legislation to encourage public education of adults and teens on ETS as well as smoking and its inadvertent effect on those who socialize with smokers will likely prevent many diseases associated with ETS exposure and improve the health of the Cambodian teens and the general population at large.
